# Advancements and recent explorations of anti-cancer activity of chrysin: from molecular targets to therapeutic perspective

**DOI:** 10.37349/etat.2024.00230

**Published:** 2024-05-23

**Authors:** Abhilasha Sood, Arpit Mehrotra, Ujjawal Sharma, Diwakar Aggarwal, Tejveer Singh, Moyad Shahwan, Ammar Abdulrahman Jairoun, Isha Rani, Seema Ramniwas, Hardeep Singh Tuli, Vikas Yadav, Manoj Kumar

**Affiliations:** The First Clinical Medical College of Lanzhou University, China; ^1^Department of Allied Health Sciences, Chitkara School of Health Sciences, Chitkara University, Rajpura 140401, India; ^2^Department of Human Genetics and Molecular Medicine, Central University of Punjab, Bhatinda 151001, India; ^3^Department of Bio-Sciences and Technology, Maharishi Markandeshwar Engineering College, Maharishi Markandeshwar (Deemed to Be University), Ambala 133207, India; ^4^Translational Oncology Laboratory, Department of Zoology, Hansraj College, Delhi University, New Delhi 110007, India; ^5^Department of Clinical Sciences, College of Pharmacy and Health Sciences, Ajman University, Ajman 346, United Arab Emirates; ^6^Centre of Medical and Bio-Allied Health Sciences Research, Ajman University, Ajman 346, United Arab Emirates; ^7^Health and Safety Department, Dubai Municipality, Dubai 67, United Arab Emirates; ^8^Discipline of Clinical Pharmacy, School of Pharmaceutical Sciences, Universiti Sains Malaysia (USM), Pulau Pinang 11500, Malaysia; ^9^Department of Biochemistry, Maharishi Markandeshwar College of Medical Sciences and Research (MMCMSR), Sadopur, Ambala 134007, India; ^10^University Centre for Research and Development, University Institute of Pharmaceutical Sciences, Chandigarh University, Gharuan, Mohali 140413, India; ^11^Department of Translational Medicine, Clinical Research Centre, Skane University Hospital, Lund University, SE 20213 Malmö, Sweden; ^12^Department of Chemistry, Maharishi Markandeshwar Engineering College, Maharishi Markandeshwar (Deemed to Be University), Ambala 133207, India; ^13^Department of Chemistry, Maharishi Markandeshwar University Sadopur, Ambala 134007, India

**Keywords:** Chrysin, anti-cancer, anti-apoptosis, anti-angiogenesis, anti-metastasis, nanoformulations

## Abstract

In recent times, there have been notable advancements in comprehending the potential anti-cancer effects of chrysin (CH), a naturally occurring flavonoid compound found abundantly in various plant sources like honey, propolis, and certain fruits and vegetables. This active compound has garnered significant attention due to its promising therapeutic qualities and minimal toxicity. CH’s ability to combat cancer arises from its multifaceted mechanisms of action, including the initiation of apoptosis and the inhibition of proliferation, angiogenesis, metastasis, and cell cycle progression. CH also displays potent antioxidant and anti-inflammatory properties, effectively counteracting the harmful molecules that contribute to DNA damage and the development of cancer. Furthermore, CH has exhibited the potential to sensitize cancer cells to traditional chemotherapy and radiotherapy, amplifying the effectiveness of these treatments while reducing their negative impact on healthy cells. Hence, in this current review, the composition, chemistry, mechanisms of action, safety concerns of CH, along with the feasibility of its nanoformulations. To conclude, the recent investigations into CH’s anti-cancer effects present a compelling glimpse into the potential of this natural compound as a complementary therapeutic element in the array of anti-cancer approaches, providing a safer and more comprehensive method of combating this devastating ailment.

## Introduction

Cancer is a complex condition caused due to abnormal growth of certain cell types, which is uncontrolled and in general leads to spread of tumor causing cells, while disturbing the normal functioning of surrounding cells. The treatment strategies for cancer are being widely explored since the foundation of the concept of healthcare itself [1, 2]. Among the available treatment options in cancer, chemotherapy is the last and final choice of the healthcare practitioners with few promising results. Comprising of molecular level changes (such as, epigenetically); the various stages of cancer offer multiple entry points where possible intervention could lead to beneficial chances of treatment/reversal in the development of cancer before it is set to enter the stage of becoming malignant [3]. Modern medications can kill tumor cells rather effectively, but they also impact the patient’s normal healthy cells and can have strange side effects. Additionally, chemotherapeutic drug resistance has constantly been a risk. Therefore, it is vitally necessary to synthesize novel chemotherapeutic medications to combat the threat of drug resistance [4]. However, in this evolving field of cancer therapy, treatment options including natural products are important to be thoroughly researched and explored for their potential application in preventing the growth and spread of cancerous cells affecting the tissue and organ functioning. Several studies targeting cancer initiation and progression have shown the potential of naturally occurring molecules as pharmacological agents targeting chemoprevention [5]. Moreover, through targeting essential components in cancer-related signaling pathways, tissue differentiation-inducing non protein coding RNA (TINCR), which has been reported to be highly dysregulated in many malignancies, influences tumour formation and progression [6]. Chrysin (CH) is one such flavonoid that has been reported to show a significant potential application in this area. It is a naturally occurring flavonoid compound found in honey, propolis, passion flowers, *Passiflora caerulea* and *Passiflora incarnata*. Flavonoids are plant-based poly-phenolic phytochemicals that are reported to have anti-cancer and chemo-preventive properties [7]. Phytochemicals such as coumarin, gallic acid’s anti-cancer activities are unaffected by a number of biological processes, including the activation of programmed cell death, cell cycle arrest, reluctance of tumour migration, and inflammation and now CH is being extensively researched for its anticancer effects and till now a plethora of evidence-based studies are predicting a mixed overview on its anticancer potential [8, 9]. Several *in vitro* and *in vivo* studies have shown potential anti-cancer effects of this flavonoid in breast cancer, prostate cancer, and lung cancer, etc. The anticancer effects of CH are accounted to its ability to interfere with the signalling pathways associated with inflammation and apoptosis (Table 1). The flavone has also been reported for interesting pharmacological properties such as anti-diabetic, immunomodulatory, anti-depressant, etc. It has been shown to induce apoptosis in cancer cell lines and inhibit angiogenesis in certain tumor types [10]. Till date the explored molecular targets of CH include: apoptotic proteins like B-cell lymphoma-2 (Bcl-2), caspases; cell cycle regulation proteins: cyclins, cyclin-dependent kinases (CDKs); phosphoinositide 3-kinases (PI3K)/protein kinase B (Akt)/mechanistic target of rapamycin (mTOR) pathway proteins; transcription factor nuclear factor-kappaB (NF-κB); reactive oxygen species (ROS) producing oxidative stress pathway proteins; angiogenesis related proteins; metastasis pathway involved in cancer cell migration and invasion; estrogen receptors (ERs) like ERβ; growth factor signaling pathway proteins like epidermal growth factor receptor, etc. [11]. The present review will focus on the molecular targets of CH in cancer research, their interpreted mechanism of action and the future prospects of utilization of CH as integration to drug development against different types of cancers (Table 2).

**Table 1:** Anticancer effects of CH based on *in vitro* studies

**Type of cancer**	**Study model**	**Effects**	**Mechanisms**	**Concentration**	**References**
Melanoma	SK-ML-28, MelC and B16F10	Anti-proliferative, inhibits angiogenesis	↓ Cancer cell proliferation, ↑ arrest in the G2/M phase, ↓ cell numbers in the G0/G1 phase, ↑ tetraploid cells, ↑ DNA damages, ↑ γ-histone 2AX (γH2AX), ↓ ataxia telangiectasia and Rad3-related (ATR), ↑ p-check point kinase1 (Chk1), ↑ p-ataxia-telangiectasia mutated (ATM), ↑ p-p53 (Ser15), ↓ vascular endothelial growth factor (VEGF), ↓ VEGF-receptor 2 (VEGFR2), ↓ hypoxia-inducible factor-1 alpha (HIF-1α), ↓ HIF-1β, ↓ p-signal transducer and activator of transcription 3 (STAT3, pY705), ↑ ROS	0 µmol, 20 µmol, 40 µmol, and 80 µmol	[12]
	A375SM and A375P	Induces apoptosis and autophagy	↓ Viability of cancer cells, ↑ nuclear and chromatin condensation, ↑ Bcl-2-associated X (Bax), ↑ c-polyadenosine-diphosphate-ribose polymerase (PARP), ↓ Bcl-2, ↑ light chain 3 (LC3), ↑ beclin 1, ↑ autophagic vacuoles, ↑ acidic vesicular organelles, ↓ p-mTOR, ↓ p-70-kDa ribosomal protein S6 kinase (P70S6K), ↓ p-S6K, ↓ p-eIF4E-binding protein (4EBP1)	0 µmol, 20 µmol, 40 µmol, 60 µmol, 80 µmol and 100 µmol	[13]
	B16F10, RAW264.7, DC2.4	Improve tumor immune response	↓ Cancer cell viability, ↑ major histocompatibility complex I (MHCI), ↑ interleukin-12 (IL-12), ↓ IL-10, ↑ STAT4, ↑ interferon-γ (IFN-γ), ↑ tumor necrosis factor-ɑ (TNF-ɑ)	-	[14]
Myeloid	Myeloid-derived suppressor cells (MDSCs), granulocytic MDSC (G-MDSC), and monocytic MDSC (M-MDSC)	Induces apoptosis	↑ G0/G1 cell cycle arrest, ↓ proliferation of MDSCs, ↓ arginine-1 (Arg-1), ↓ cyclooxygenase-2 (COX-2), ↓ inducible nitric oxide synthase (iNOS), ↓ nitric oxide (NO), ↑ p-Akt	10 µmol (low dose) and 20 µmol	[15]
Leukemia	U937	Induces apoptosis	↑ Bax, ↓ Bcl-2, ↑ caspase-3, ↑ phospholipase C-1 degradation, ↓ X-linked inhibitor of apoptosis protein (XIAP), ↓ Akt	-	[16]
Mucoepidermoid	MC-3	Induces apoptosis and autophagy	↓ Cancer cell viability, ↑ nuclear condensation and shrinkage, ↑ apoptotic bodies, ↑ c-PARP, ↑ Bax, ↓ Bcl-2, ↑ LC3-II, ↑ beclin 1, ↓ sequestosome 1 (p62), ↓ p-mTOR, ↓ p-extracellular signal-regulated kinases 1/2 (ERK1/2), ↑ p-Jun N-terminal kinase (JNK), ↑ p-p38	0 µmol, 50 µmol, and 100 µmol	[17]
Nasopharyngeal carcinoma	CNE1	Induces apoptosis	↑ Cancer cell death, ↑ sub-G1 population, ↑ cell shrinkage, ↑ chromatin condensation, ↑c-PARP cleavage, ↑ c-caspase 8	10 µmol, 20 µmol, and 40 µmol	[18]
Brest cancer	MDA-MB-231	Induces apoptosis	↓ HIF-1α, ↑ Bax, ↑ p53, ↓ Bcl-2, ↓ cyclin D1, ↓ p-STAT3	CH and radiotherapy, combination index (CI) of 0.495	[19]
	T47D	Induces apoptosis	↑ Cytotoxicity towards cancer cell, ↓ *hTERT* gene	Artemisinin and CH encapsulated poly(lactic-co-glycolic acid) (PLGA)-poly(ethylene glycol) (PEG) nanoparticles (NPs), IC_50_ = 12.51 μM	[20]
	Breast and BT474	Induces apoptosis and anti-proliferative	↑ Destabilization of the genome, ↓ cancer cell survival, ↑ γH2AX, ↑ PKcs-pS2056, ↓ p53-binding protein 1 (53BP1), ↓ RAD51	0 µmol, 5 µmol, 10 µmol, 15 µmol, 20 µmol, and 30 µmol	[21]
	MDA-MB-231 and MDA-MB-231_luc cells	Inhibits metastasis	↓ Cancer cell viability, ↓ PI3K, ↓ NF-κB, ↓ matrix metalloproteinases-10 (MMP-10), ↓ MMP-2	0–100 µmol	[22]
	MDA-MB-231	Anti-proliferative and induces apoptosis	↑ Cytotoxicity towards cancer cell, ↑ cell accumulation in G2/M phase, ↑ apoptosis frequency, ↑ microRNA-132 (miR-132), ↑ miR-502c, ↓ HN1 and P65	Curcumin and CH encapsulated PLGA-PEG NPs, combination index (CI_50_) = 0.47	[23]
	MCF7	Induces apoptosis	↓ Cancer cell viability, ↑ apoptosis frequency, ↑ apoptotic cell bodies	5 mmol/L, 10 mmol/L, 20 mmol/L, and 30 mmol/L	[24]
Lung	A549	Induces apoptosis	↓ Cancer cell viability, ↑ caspase-3 and caspase-9, ↑ Bax/Bcl-2 artio, ↑ Bax, ↓ Bcl-2	IC50 = 49.2 (48 h) and 38.7 (72 h)	[25]
Gastric	AGS	Inhibits metastasis	↓ Endogenous and inducible receptor originated from nantes (RON) expression, ↓ growth response-1 (Egr-1), ↓ NF-κB, ↓ phorbol-12-myristate-13-acetate-(PMA)	0–100 µmol	[26]
	AGS	miRNA related	↑ Cytotoxic towards cancer cell line, ↑ miR-9, ↑ Let7-a, ↓ miR-18a, ↓ miR-21, ↓ miR-221	PLGA-PEG encapsulated CH (0–160 µmol)	[27]
Hepatoma	HepG2	Induces apoptosis	↑ Cancer cell death, ↑ sub-G1 population, ↑ cell shrinkage, ↑ chromatin condensation, ↑ c-PARP cleavage, ↑ c-caspase 8	10 µmol, 20 µmol, and 40 µmol	[18]
Pancreatic	MIA PaCa-2	Induces apoptosis	↓ Cancer cell viability, ↑ caspase-3, ↑ c-PARP, ↑ G protein-coupled estrogen receptor, ↑ G2/M phase cells, ↓ ERα, ↓ c-Myc	0–100 µmol	[28]
Colorectal	SW48, SW480, SW620, HT-29 and HCT-116	Induces autophagy	↓ Cancer cell viability, ↑ LC3II, ↓ p-mTOR, ↑ p-adenosine monophosphate-activated protein kinase (AMPK), ↓ p-Akt, ↑ ROS production	0 µmol, 20 µmol and 50 µmol	[29]
	HCT-116	Induces apoptosis	↑ Cancer cell death, ↑ sub-G1 population, ↑ cell shrinkage, ↑ chromatin condensation, ↑ c-PARP cleavage, ↑ c-caspase 8	10 µmol, 20 µmol, and 40 µmol	[18]
Prostate	PC-3	Induces apoptosis	↓ Vasculogenic mimicry (hypoxia-induced) ↓ HIF-1α, ↓ vascular endothelial-cadherin (VE-cadherin), ↓ VEGF, ↓ Bcl-2, ↑ c-PARP, ↑ caspase-3, ↓ sphingosine kinase-1 (SPHK-1), ↓ p-Akt, ↓ p-glycogen synthase kinase 3β (GSK-3β)	10 µmol	[30]
	PC-3	Induces apoptosis	↓ Cancer cell viability, ↑ cell shrinkage, ↑ apoptosis frequency	10 µmol, 20 µmol, 30 µmol, and 40 µmol	[31]
Endometrial	HEC1Aand Ishikawa	Induces autophagy and apoptosis	↓ Proliferation and colony formation activity, ↑ apoptotic cells, ↑ Bax, ↓ Bcl-2, ↑ LC3II, ↑ Beclin 1, ↓ p62, ↑ ROS accumulation, ↓ pAkt, ↓ pmTOR	0 µmol, 10 µmol, 20 µmol, 40 µmol, and 80 µmol	[32]
Cervix	Hela	Induces apoptosis	↑ Cancer cell death, ↑ sub-G1 population, ↑ cell shrinkage, ↑ chromatin condensation, ↑ c-PARP cleavage, ↑ c-caspase 8	10 µmol, 20 µmol, and 40 µmol	[18]

↑: over expression; ↓: down expression; -: blank

**Table 2:** Anticancer effects of CH based on *in vivo* studies

**Type of cancer**	**Animal models**	**Effects**	**Mechanisms**	**Dosage**	**Duration**	**References**
Melanoma	C57BL/6JRj mice xenografted with B16F10	Inhibits angiogenesis	↓ Tumor volume, ↓ vascular segments, ↓ vascular network length, ↓ VEGF-A, ↓ phosphorylated histone H2AX on Serine 139 (pS139-H2AX), ↓ HIF-1α, ↓ Nrf1, ↓ superoxide dismutase 1 (Sod1), ↓ peroxiredoxin 4 (Prdx4), ↓ glutathione peroxidase (Gpx)	50 mg/kg	16 days	[12]
	C57BL/6 mice xenografted with B16F10	Improve tumor immune response	↓ Cancer cell growth in tumors, ↑ survival rate in mice↑ vaccine efficacy, ↑ cluster of differentiation 80 (CD80), ↑ CD86, ↑ MHCI, ↑ MHCII, ↑ CD8^+^ T cells	650 mg/kg	21 days	[14]
	C57BL/6 mice xenogafted with B16F10	Inhibits angiogenesis and inhibited tumor growth	↓ Tumor volume and weight, ↓ G-MDSCs buildup in marrow and spleen, restore T cell proliferation, ↓ activation of RhoA, ↓ HIF-1α, ↓ microvessel density, ↓ vascular permeability ↑ vascular perfusion	20 mg/kg and 40 mg/kg	22 days	[15]
Breast	BALB/c nude mice xenogafted with MDA-MB-231	Induces apoptosis, inhibits metastasis	↓ Tumor growth, ↓ Ki-67, ↑ apoptosis frequency, ↓ MMP-1, 2, 3, 9, 10, and 13, ↑ MMP-8, ↓ tissue inhibitor of metalloproteinase-1 (TIMP-1), ↓ PI3K, ↓ p-Akt, ↓ GSK-3β, ↓ NF-κB	CH-NPs—10 mg/kg	21 days	[22]
Pancreatic	BALB/c-nude mice xenogarfted with MIA PaCa-2	Induces apoptosis	↓ Tumour growth, ↓ Ki-67, ↓ c-Myc	50 mg/kg	35 days	[28]
Prostate	Male BALB/c nude mice xenograted with PC-3 cells	Induces apoptosis	↓ Tumor spheroid formation and growth, inhibited PC-3 tube formation, ↓ HIF-1α, ↓ VE-cadherin (under normoxic as well as hypoxic conditions), ↓ Ki-67, ↓ SPHK-1, ↓ cyclin D1, ↓ c-caspase-3, ↓ VEGF	50 mg/kg	25 days	[30]

↑: over expression; ↓: down expression

## Chemistry of CH

CH is a flavone with the chemical formula 5,7-Dihydroxyflavone or 5,7-Dihydroxy-2-phenyl-4H-1-benzopyran-4-one. It has a 15-carbon polyphenolic skeleton called as flavonoid. Structurally this flavonoid consists of two benzene rings (A, B) and an oxygen-containing ring (C). CH possesses a 2 to 3 carbon double bond and a carbonyl group at carbon 4. Additionally, two hydroxyl groups are present at carbons 5 and 7 [33]. The chemical formula of CH is C_15_H_10_O_4_ and it has a molar mass of 254.241 mol/g. Unique to this flavone is the presence of C2-C3 double bond in ring C and the absence of oxygenation at C-3, in comparison to the other flavonoids that have either C-3 or C-4 di-ortho hydroxyl groups in the ring-B while CH shows absence of oxygenation in this particular ring. The other molecular derivatives of CH like wogonin, baicalein and oroxylin A are known to be formed by the oxygenation of ring A of the molecule. In comparison to other flavonoids the absence of oxygenation at the B and C rings are considered to be one of the major contributing factors in the antioxidant and anti-inflammatory activitiy of this flavone [34].

## CH mediated apoptosis induction and cell cycle arrest

**Figure 1:**
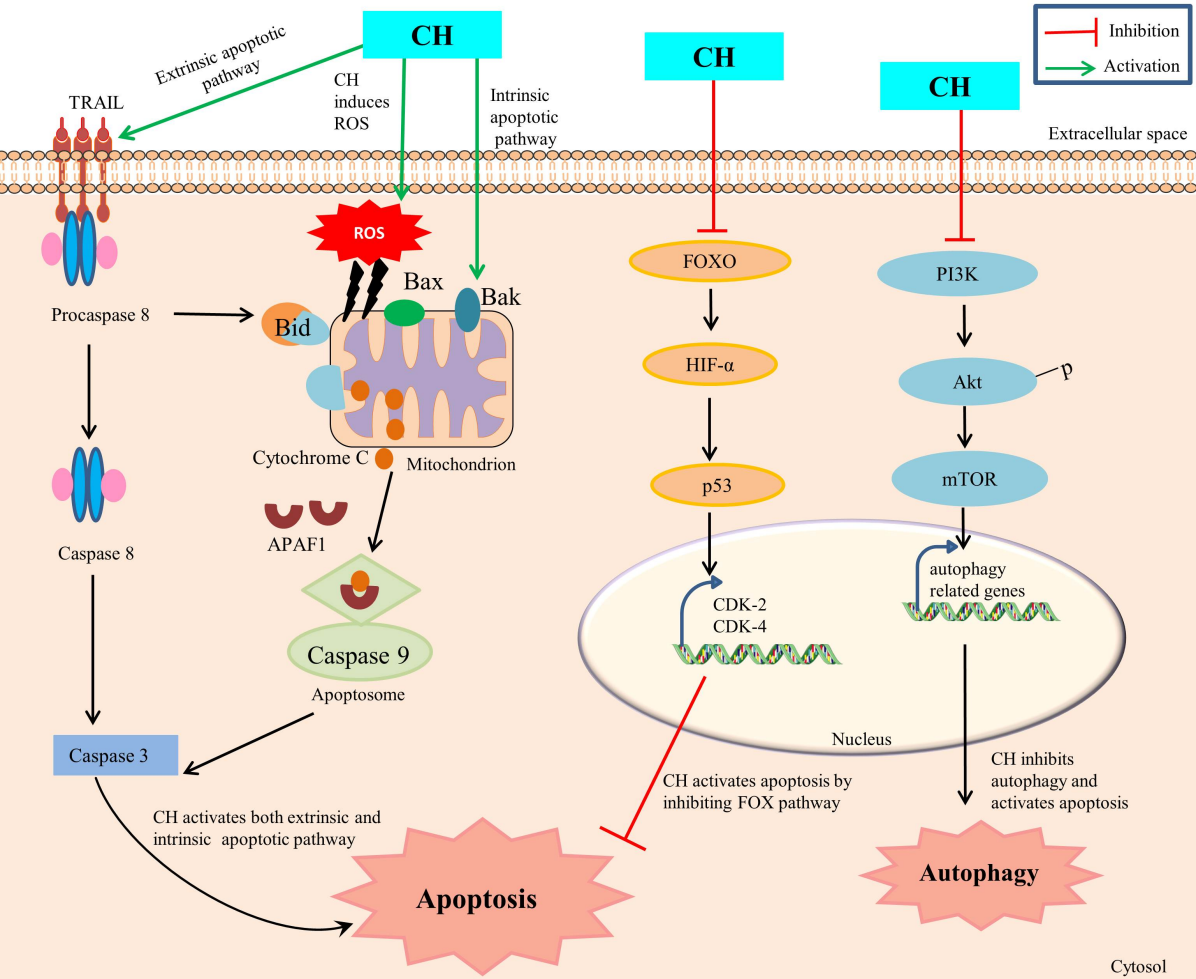
CH mediated apoptosis and autophagy. TRAIL: TNF-related apoptosis-inducing ligand; APAF1: apoptotic protease activating factor-1; Bid, Bax, Bak: pro-apoptotic factors

The CH mediated mechanism of apoptosis initiation in cancer cells is a collection of numerous pathways with precise involvement of several molecular partners, though the ensuing of a specific mechanism depends on cancer cell types. The explored key mechanisms involve: activation of mitochondria-dependent intrinsic pathway, caspase activation, regulation of Bcl-2 Family of proteins, modulation of cell survival signaling pathways, induction of oxidative stress and regulation of apoptotic regulators [35] (Figure 1). Initially, CH prompts apoptosis induction by influencing the intrinsic mitochondrial pathway via interrupting the stable existence between pro-apoptotic (Bax, Bak) and anti-apoptotic [Bcl-2, Bcl-extra large (Bcl-xL)] proteins within the mitochondrial membrane. Such a disproportion causes permeabilization of outer membrane mitochondria, with concomitant releasing of cytochrome c and other apoptogenic proteins from the mitochondrial environment into the cytoplasm, consequently ensuing apoptosis [36]. Members of the Bcl-2 family, which is frequently increased in human malignancies, include the anti-apoptotic proteins Bcl-2 and myeloid cell leukemia 1 (Mcl-1), which are linked to chemotherapeutic resistance and relapse. The cleavage and inactivation of Mcl-1 by caspase-3 on the onset of apoptosis amplifies the cascade of caspase activation. Caspase-8 is activated by pro-apoptotic extracellular signaling, which cleaves and activates Bid, which binds to and inhibits Mcl-1. In order to maintain the integrity of the mitochondria, Mcl-1 uses its antiapoptotic activity to combat the activation of Bax and Bak [37]. Moreover, CH has shown the propensity to obstruct cell survival signaling pathways that are often observed to be hyperactive during autophagy in cancer cells. As such, CH can compromise the normal working of PI3K/Akt and MAPK/ERK pathways (foremost therapeutic targets in cancer treatment) by suppressing mTOR expression, thus inhibiting autophagic thrust with simultaneous enhancement of apoptosis progression in cancer cells [13]. In addition, CH can also manipulate the expression and regulation of other apoptotic partner activities such as by enhancing the p53-mediated apoptosis in response to DNA damage with simultaneous inhibition of CDK2 and CDK4, forkhead box O (FOXO) pathway and HIF-1α expression. This CH mediated disruption has been reported to interrupt cellular signals which prompt cells to move from one phase of the cell cycle to the next and thus preventing cancer cells from dividing any further [38]. Furthermore, CH is reported to promote the production of reactive oxygen species which leads to generation of oxidative stress within cancer cells, and as a consequence such enhanced levels of reactive species are prone to damage cellular components like DNA, proteins and lipids in cancer cells [29].

Since such mechanisms are highly intricate, therefore a precise mechanism of CH mediated apoptosis induction is suggested to differ based on specific cancer type, genetic constitutes of cells and the concentration of CH administered. Ongoing investigations foster deeper understanding of such mechanisms in order to substantially exploit the apoptotic properties of CH for therapeutic interventions.

## Anti-angiogenesis and anti-metastasis properties of CH

CH has been extensively studied for its distinguished anti-angiogenesis and anti-metastasis properties in context to treating cancer. Since angiogenesis is fundamental for proliferation of its associated tumorigenicity in affected tissues, the inhibitory potential of CH in such circumstances has been well reported to be mediated through various mechanisms such as: inhibition of VEGF and MMPs, modulation of signaling pathways and anti-metastasis properties, regulation of epithelial-mesenchymal transition (EMT), and anti-inflammatory effects. VEGF is a key regulatory protein involved in promoting angiogenesis and CH has been reported to inhibit the expression and simultaneous inactivation of VEGF, thereby limiting the augmentation of new blood vessels leading to restricted blood supply to cancer cells [39]. The ability of cancer cells to invade surrounding tissues and promote angiogenesis is highly dependent on MMPs activity and CH has been shown to significantly inhibit such activation, consequently hampering MMPs (MMP-9) expression by suppressing AP-1 activity via blocking JNK/c-Jun and ERK/c-Fos signaling pathways in cancer cells [40]. The PI3K/Akt/mTOR pathway, besides being involved in regulating apoptosis is also implicated in promoting angiogenesis in cancer cells and CH intervention is suggested to obstruct such pathways efficiently, thereby limiting their capacity to establish secondary tumors in distant organs [12, 15]. Moreover, CH has shown to inhibit hypoxia-driven progression of vasculogenic mimicry during angiogenesis, which is a common characteristic feature observed in tumour microenvironments by reducing the expression of HIF-1α, SPHK-1 and phospho-Akt/GSK-3β signaling in cancer cells [30].

**Figure 2:**
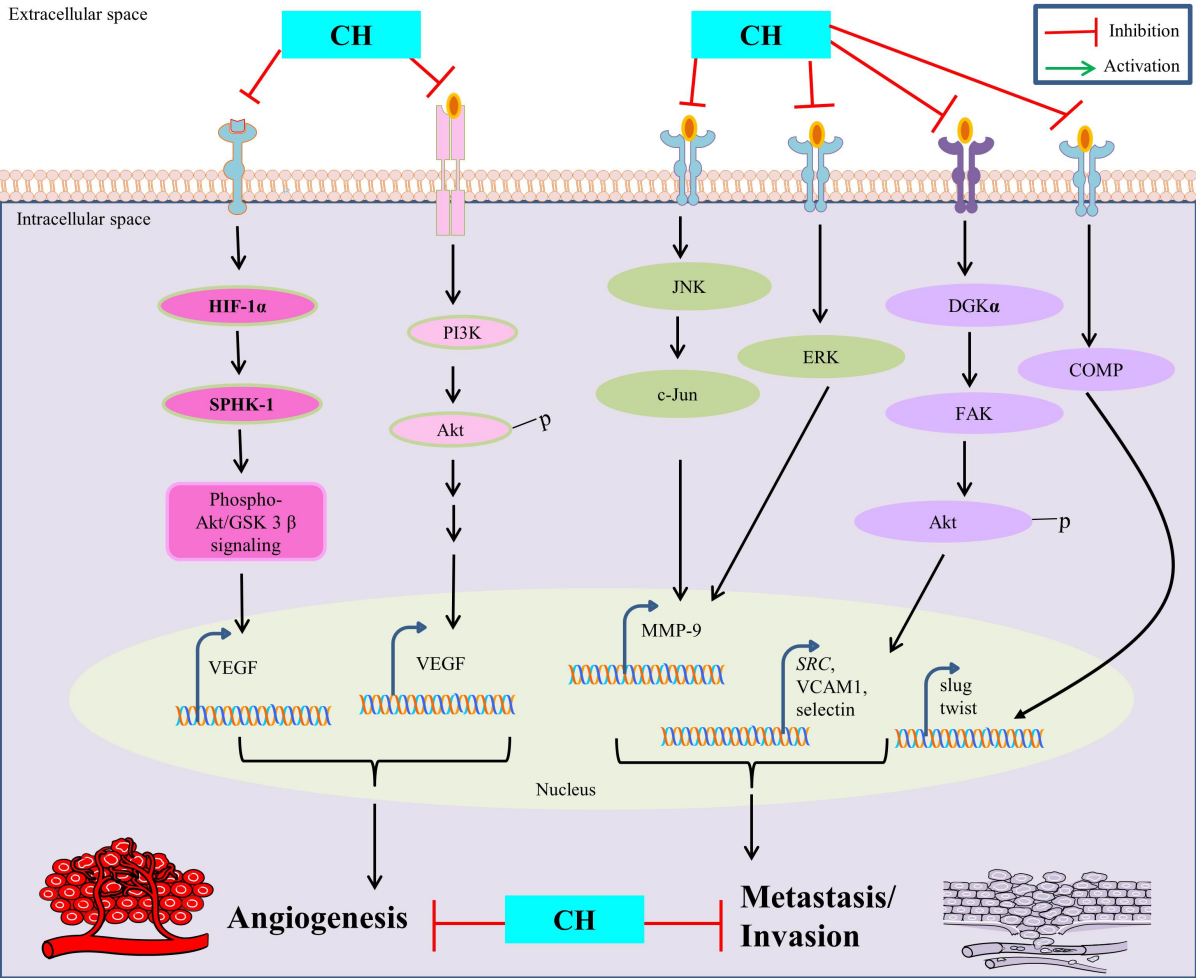
Anti-angiogenetic and anti-metastatic properties of CH. *SRC* is also known as proto-oncogene c-Src. VCAM1: vascular cell adhesion molecule 1

However, anti-metastasis properties of CH are mainly attributed to its ability to inhibit cell migration and invasion in surrounding tissues. This is achieved by disrupting the assembly of diacylglycerol kinase-α (DGKα)/focal adhesion kinase (FAK) signalosome with concomitant inhibition of FAK/Akt pathway involved in cell adhesion and mobility, thus reducing adhesion of cancer cells to extracellular matrix and their susceptibility to proliferate further [41]. CH is also involved in modulating EMT, a process wherein cancer cells lose their epithelial features to gain mesenchymal characteristics, thus enabling them to migrate and invade. CH can suppress such EMT related factors (like snail, slug, and twist) via inhibition of cartilage oligomeric matrix protein (COMP) expression and helps to maintain cancer cells in a less invasive state, thus considered as a potential therapeutic target [42] (Figure 2).

In addition, CH ability to modulate immune response has shown to potentially influence dynamic interactions between cancer cells and immune cells within the tumour micro-environment, thus limiting the ability of tumors to metastasize. Furthermore, additional investigations are required to strengthen our understanding of CH efficacy for offering such defensive mechanisms and their clinical applications.

## Anti-inflammatory mechanisms

**Figure 3:**
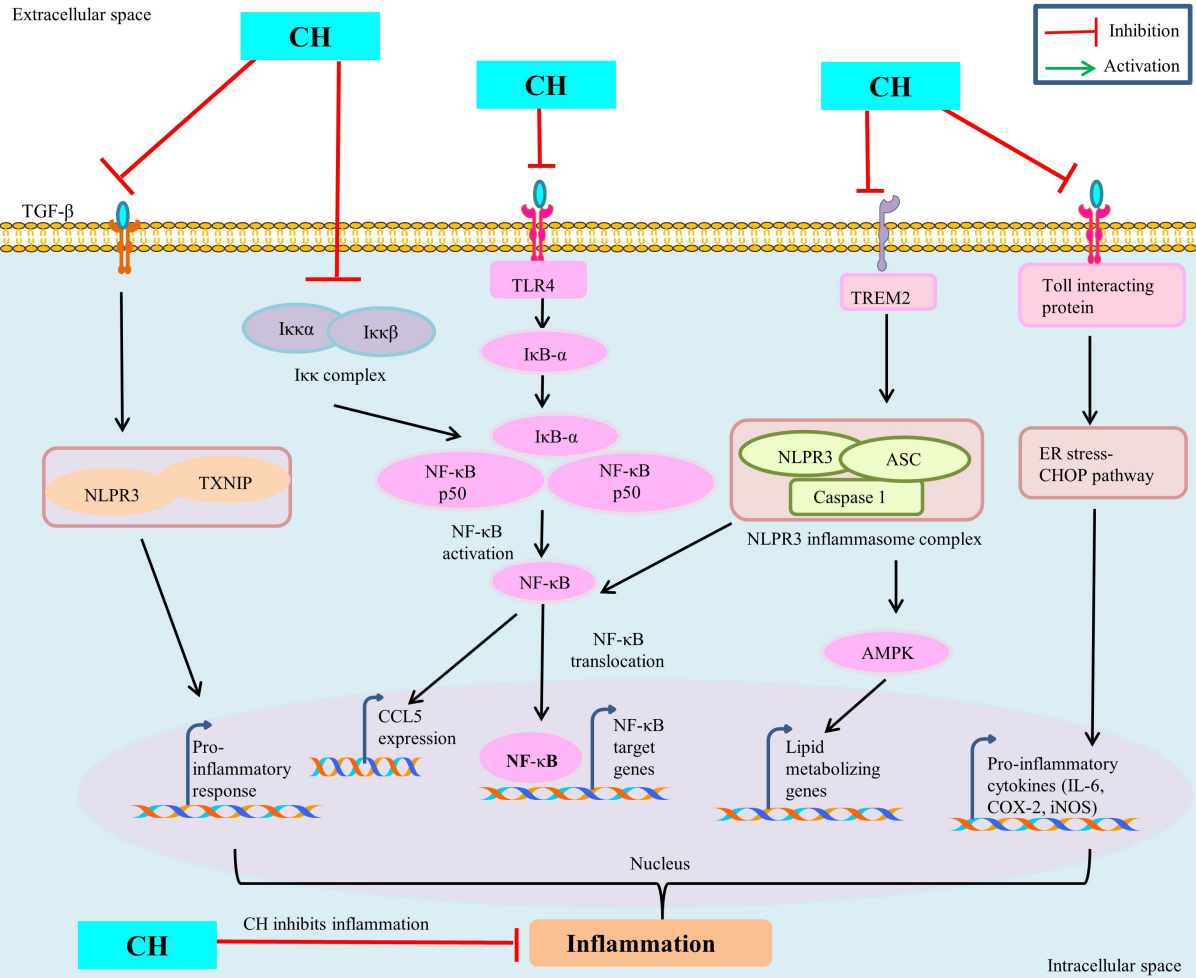
Anti-inflammatory mechanism of CH. TREM: triggering receptors expressed on myeloid cells

The seventh cancer-related hallmark identified is inflammation [43]. CH has been demonstrated to have a number of advantageous pharmacological effects including anti-cancerous properties through modulating signaling pathways involved in inflammation [11]. Numerous *in vivo* and *in vitro* models have shown the anti-inflammatory properties of CH. Numerous studies have looked at *in vitro* inflammation in lipopolysaccharide (LPS)-treated murine macrophages (Raw 264.7). CH derivative (CM1) inhibited LPS-induced expression of pro-inflammatory cytokines IL-6 and TNF-α, COX-2, and iNOS-mediated NO by up-regulating toll-interacting protein expression [44] and via the ER stress-CHOP pathway in polyinosinic-polycytidylic acid -induced macrophages [45]. CH has antioxidant properties and lowers the macrophage inflammatory protein (MIP), MIP-2, MIP-1α, and MIP-1β production [45]. Further, CH treatment down regulate the inflammatory mediators like IL-6, COX-2, TNF-α, NF-κB, iNOS and prostaglandin E2 (PGE2) in ferric nitrilotriacetate induced renal cancer [46, 47]. CH prevented C-C chemokine ligand 5 (CCL5) expression by targeting the inhibitor of κB (IκB) kinase (IKK), via binding to its ATP-binding pocket subsequently, prevent IκB degradation and NF-κB activation and thus attenuate the inflammatory responses [48] (Figure 3). By modifying the immune system’s response to various stimuli, inflammasomes play a significant part in tumorigenesis. It has been demonstrated that nucleotide-binding oligomerization domain and leucine-rich repeat and pyrin domain-containing 3 (NLRP3) inflammasome upregulation contributes to tumour development and metastasis in a variety of human cancers [49]. Inflammation is prevented by CH treatment because it decreased lipid metabolism by inhibiting AMPK and inflammasome activation by blocking NLRP3 [50]. CH may treat inflammation and prevent fibrosis by decreasing the inflammatory response and fibrosis induced by TGF-β1 in synovial fibroblasts (SFs) via inhibiting thioredoxin-interacting protein (TXNIP)-NLRP3 interactions in TGF-β-induced SFs [51]. CH reduces hypercholesterolemia-mediated atherosclerosis via modulating the inflammatory genes expression including *TNF-α*, toll-like receptors (*TLR4*), *IL-17*, and *NLRP3* in the intestine and aorta compared with hypercholesterolemia control rats [52]. ERK1/2, a type of extracellular regulated kinase that is found to be activated in a number of cancers [53] regulates a range of processes from metabolism to inflammation [54]. Moreover, CH was found to be linked with reduced p-ERK/ERK and p-Akt/Akt protein expression in colorectal cancer cells SW620 [33, 55]. These findings imply that CH has anti-inflammatory mechanisms against inflammatory diseases and may be helpful in the treatment of chronic inflammatory diseases like cancer.

## Nanoformulation of CH

Natural flavone CH, which has anti-inflammatory and anticancer properties without harming healthy cells or having any negative side effects, is a standout candidate for promoting health benefits [33]. Despite the fact that CH has been shown to be effective in treating cancer, there are significant obstacles that prevent wider clinical application. CH has unfavourable properties that can limit its therapeutic effects, including low physicochemical stability, poor solubility in water, rapid hepatic and intestinal metabolism, and low cellular uptake [56, 57]. A promising approach to enhance compound delivery to cancer cells while assisting in increased bioavailability and reduced *in vivo* degradation rate is to use nanotechnological methods that allow slow, sustained, and controlled release of the encapsulated agents. Therefore, the properties and anticancer efficacy of such active substances can be enhanced by encapsulating them in shells (liposomes, NPs, etc.). More often than not, polymeric NPs outperform liposomes in terms of size distribution, stability, harmonic physicochemical features, sustained and controllable drug-release profiles, and loading capacities for weakly water-soluble agents [58]. Polymeric NPs have attracted remarkable interest from academia, the medical community, and business due to these distinctive qualities. Several natural polymers, including polysaccharides and chitosan, as well as a number of Food and Drug Administration (FDA)-approved synthetic materials, including polycaprolactone (PCL), PLGA, and PEG, have all been extensively studied for NP synthesis [59–61]. The two primary techniques for creating PEG-PLGA NPs are nanoprecipitation and double emulsion-solvent evaporation; both make use of the self-assembly of PEG and PLGA at a particular temperature and ratio [62]. When compared to free CH, CH-loaded PEG-PLGA NPs had higher solubility and growth-inhibitory activity against AGS cells and upregulated miR-34a [63]. Due to increased uptake by gastric cancer cells, CH-PLGA-PEG NPs were more effective than pure CH in upregulating miR-9 and Let-7a [27]. Additionally, PEG-PLGA NPs co-loaded with 5-fluorouracil or curcumin showed substantial synergistic anticancer effects in the treatment of colorectal cancer [64, 65].

CH has also several drawbacks that limit its biological function, such as a short half-life, limited solubility, poor biological availability, a short circulatory stability length, and fast metabolism and degradation [66]. However, improvements in nanotechnology have considerably mitigated these drawbacks. To improve the water solubility of CH, a colloidal delivery system comprising nanoemulsion (NE) was made as CH-NE using an oil-in-water system. Results showed that the solubility of CH has increased to 160 µg/g when compared to the parent form at 20 µg/g [67]. Chemical conjugation with other compounds has been performed to improve CH’s functions. The anti-inflammatory function of CH is elevated when conjugated with indole and barbituric acid [68]. CH has the ability to increase the expression of miR-9, an onco-suppressor factor in gastric cancer. The use of NPs can significantly increase miR-9 expression compared to pure form [27]. CH and docetaxel-loaded micelles can increase cytotoxicity in tumor cells. They can increase ROS levels and cause accumulation as micelles [69]. NPs can also act as carriers for the co-loading of CH with other phytochemicals or anti-tumor compounds, such as curcumin. This combination provides a synergistic response and maximizes the cytotoxicity of these phytochemicals against tumor cells [64]. Polymeric NPs are also found to enhance cellular uptake of CH in breast cancer treatment. CH-loaded polymeric NPs have shown targeted delivery of CH at tumor sites, leading to increased anti-tumor activity [70]. In general, to overcome the conventional drawbacks of CH, such as poor bioavailability, rapid metabolism and elimination, and a short half-life, which all limit its therapeutic efficacy, researchers are testing the use of nanocarriers or NPs to encapsulate CH, which can meet all the limitations and improve its targeted delivery, thereby improving therapeutic efficacy.

## Challenges in delivery

CH is a naturally occurring compound that has shown potential anticancer effects through selective cell signaling mechanisms. Anyhow, its delivery to target tissues in cancer treatment faces challenges, partly due to its low bioavailability, poor solubility, short half-life, and rapid metabolism and degradation [71]. The poor solubility of CH in water and under physiological conditions has been recognised as a primary challenge that restricts its bioavailability and therapeutic efficacy. Various pharmaceutical studies have demonstrated that the oral bioavailability of CH is very poor, less than 1%, due to its low aqueous solubility and extensive metabolism [72]. Recent data demonstrates that poor absorption, rapid metabolism, and systemic elimination are responsible for poor bioavailability of CH in humans, which subsequently restricts its therapeutic effects [73]. CH is rapidly metabolized and eliminated in the human body, leading to its low bioavailability [74]. In cells like hepatocytes and enterocytes, through sulfation and glucuronidation reactions, CH is highly biotransformed into the body, resulting in the formation of conjugated metabolites such as CH sulfonate and glucuronide [75]. This all accounts for the short half-life of CH. In a recent study after the oral administration of a 400 mg single dose of CH to healthy human subjects, the peak plasma concentration of the parent compound was only 16–63 nmol, whereas its metabolites appeared approximately at 400–800 nmol concentrations in the circulation [74]. CH’s metabolites may not have the same biological activity as the parent compound [33]. Therefore, CH’s poor stability in the body and bloodstream is a significant factor in its poor efficiency. Many attempts have been made over the past years to overcome these major drawbacks by using nanoemulsions, nanosuspensions, micronization [76], nanomicelles, host-guest complexes, and different formation approaches [77]. Among these uses, the nanotechnological approach is found to be a promising one that enhances compound delivery to cancer cells while assisting in increased bioavailability and reducing *in vivo* degradation rates.

## Safety concerns

While CH shows promise as a potential therapeutic option for cancer treatment, there are safety concerns that need to be addressed before it can be widely used in clinical settings. One significant challenge with CH is its limited bioavailability, meaning the extent to which it can be absorbed and utilized by the body [78]. CH has low water solubility and undergoes rapid metabolism in the liver, which can lead to poor systemic distribution. This limits its efficacy and raises concerns about whether sufficient levels of CH can reach target tissues to exert anticancer effects [73, 78]. It is generally considered safe when consumed through dietary sources like fruits, vegetables, and honey. However, at high concentrations, it may have cytotoxic effects on healthy cells along with cancer cells. Finding the right balance between an effective dose against cancer cells and minimal harm to healthy cells is critical. CH’s potential interactions with other drugs also pose safety concerns [79, 80]. It can inhibit certain enzymes in the liver that are responsible for metabolizing various medications. This inhibition could lead to altered drug concentrations in the body, potentially resulting in adverse effects or reduced efficacy of other medications [80]. Geisen and Sturla have extensively reviewed a few examples of such drug-drug interaction problem with CH intake [81]. Furthermore, CH’s ability to modulate hormone-related pathways is considered both a potential benefit and a concern. It has been found to have estrogenic and anti-estrogenic effects, which could affect hormone-sensitive cancers [82]. These effects need to be carefully studied, as they might interact with hormonal therapies commonly used in treatment of breast and prostate cancer. It is to be noted that individuals can vary in their response to natural compounds like CH due to genetic, metabolic, and physiological differences. This variability can impact both the effectiveness and safety of CH as a therapeutic option. Several studies have reported the neuroprotective role of CH against xenobiotics, however, limited research exists on the long-term safety of CH supplementation [83, 84]. The potential cumulative effects of CH on various organs and systems over extended periods of use are not well understood. While preclinical studies have shown promising results, there is a lack of well-designed human clinical trials evaluating CH’s safety and efficacy. Therefore, rigorous clinical research is essential to establish the appropriate dosage, formulation, and overall safety profile of CH in cancer patients.

At the end, while CH holds potential as an anticancer therapeutic, its safety concerns, particularly regarding bioavailability, toxicity, drug interactions, and long-term effects, necessitate thorough investigation. Before CH can be considered a viable and safe treatment option for cancer patients, comprehensive clinical trials are essential to determine optimal dosing, administration methods, and its overall risk-benefit profile in a clinical context.

## Conclusions

In conclusion, this manuscript sheds light onto the multifaceted potential of CH as a promising candidate for combating cancer through various mechanisms. The exploration of CH’s effects, as outlined in the different sections, reveals its remarkable therapeutic attributes and provides a foundation for further research in the field of oncology. CH’s intricate chemistry discussed in the present review serves as a testament to its bioactive properties. Its ability to induce apoptosis offers a powerful tool in the fight against cancer by promoting programmed cell death in malignant cells, ultimately inhibiting their uncontrolled growth. Additionally, the anti-angiogenic effects of CH hold significant promise in curbing tumor progression by thwarting the formation of new blood vessels that nourish tumors. Furthermore, the manuscript highlights CH’s potential in combating metastasis, a critical factor contributing to cancer’s lethality. By impeding the invasion and migration of cancer cells, CH could play a pivotal role in limiting the spread of the disease to other tissues and organs. Moreover, the anti-inflammatory effects of CH underscore its role in modulating the tumor microenvironment, potentially creating an inhospitable milieu for tumor growth and progression.

One of the notable advancements discussed here involves the nano formulation of CH for targeted delivery. This innovative approach holds promise in enhancing the specificity and efficacy of CH, minimizing potential off-target effects and maximizing its therapeutic impact on cancer cells. Presumably, several challenges and safety concerns must be addressed before CH can be considered a viable therapeutic option in clinical practice. Considering individual variations in response to CH is crucial. Personalized medicine approaches, based on patients’ genetic and metabolic profiles, could help identify those who might benefit most from CH treatment and mitigate potential adverse effects. Clinical trials, translational research, and investigations into potential synergistic effects with existing treatment modalities are avenues that warrant exploration. In summation, this manuscript underscores CH’s potential as a multi-pronged approach to cancer treatment. Collective evidence discussed in this manuscript paints a picture of CH as a multifaceted warrior in the battle against cancer. As the scientific community forges ahead, building upon these findings, CH’s potential to reshape the landscape of cancer treatment becomes an exciting and hopeful prospect.

## Abbreviations

Akt: protein kinase B
Bcl-2: B-cell lymphoma-2
CDKs: cyclin-dependent kinases
CH: chrysin
COX-2: cyclooxygenase-2
EMT: epithelial-mesenchymal transition
ERK1/2: extracellular signal-regulated kinases 1/2
GSK-3β: glycogen synthase kinase 3β
HIF-1α: hypoxia-inducible factor-1 alpha
IL-12: interleukin-12
iNOS: inducible nitric oxide synthase
MIP: macrophage inflammatory protein
miR-132: microRNA-132
MMP-10: matrix metalloproteinases-10
mTOR: mechanistic target of rapamycin
NF-κB: nuclear factor-kappaB
NLRP3: nucleotide-binding oligomerization domain and leucine-rich repeat and pyrin domain-containing 3
NPs: nanoparticles
PEG: poly(ethylene glycol)
PI3K: phosphoinositide 3-kinases
PLGA: poly(lactic-co-glycolic acid)
ROS: reactive oxygen species
SPHK-1: sphingosine kinase-1
STAT3: signal transducer and activator of transcription 3
TNF-ɑ: tumor necrosis factor-ɑ
VEGF: vascular endothelial growth factor

## Declarations

### Copyright

© The Author(s) 2024.
